# A new species of the rare, deep-sea polychaete genus *Benthoscolex* from the Sea of Kumano, Japan (Annelida, Amphinomidae)

**DOI:** 10.3897/zookeys.738.22927

**Published:** 2018-02-19

**Authors:** Naoto Jimi, Taeko Kimura, Akito Ogawa, Hiroshi Kajihara

**Affiliations:** 1 Department of Natural History Sciences, Graduate School of Science, Hokkaido University, Kita 10 Nishi 8 Kitaku, Sapporo, Hokkaido 060-0810, Japan; 2 Graduate School of Bioresources, Mie University, 1577 Kurimamachiya-cho, Tsu, Mie 514-8507, Japan; 3 Graduate School of Science, The University of Tokyo, 7-3-1 Hongo, Bunkyo-ku, Tokyo 113-8654, Japan

**Keywords:** Amphinomida, deep sea, new genus record, Polychaeta, polychaetes, taxonomy

## Abstract

A new species of amphinomid polychaete, *Benthoscolex seisuiae* **sp. n.**, is described from the Sea of Kumano, Japan, from depths of 487–596 m. The species is distinguishable from its congeners by the following features: i) palps 1.8 times as long as lateral antennae; ii) branchiae do not reach to the tip of the notochaetae. This is the first record of *Benthoscolex* from Japan. A partial mitochondrial cytochrome *c* oxidase subunit I gene sequence from the holotype of *B. seisuiae* **sp. n.** is provided for reliable species identification in the future.

## Introduction

Marine annelids in the family Amphinomidae are commonly known as fireworms, characterized by having defensive, dorsally-oriented, calcareous chaetae that are thought to be used to inject a venomous substance into predators (Verdes et al. 2017). The family consists of approximately 180 nominal species in 22 genera (Borda et al. 2012; Barroso et al. 2017; Sun and Li 2017), mostly distributed in shallow and tropical waters (Barroso et al. 2017).

One genus, *Benthoscolex* Horst, 1912, is rare and known mainly from deep-sea substrates. Previous collection records of the genus are limited to low latitude areas (Horst 1912; Monro 1937; Hartman 1942; Fauvel 1953; Salazar-Vallejo 1999; Wehe and Fiege 2002). The genus consists of two species: *B. coecus* Horst, 1912 and *B. cubanus* Hartman, 1942. Previous studies have reported *B. coecus* from the Red Sea to the SW Pacific Ocean (Horst 1912; Monro 1937; Fauvel 1953; Wehe and Fiege 2002) and *B. cubanus* from the Caribbean Sea (Hartman 1942). The main diagnostic features of the genus are: 1) caruncle consisting of three ridges and 2) branchiae are absent at least in the first five chaetigers. In Japan, several amphinomids have been reported from the deep sea (e.g., Imajima 2001, 2005, 2006, 2011), but there is no record of *Benthoscolex* species from Japanese waters.

During the research cruise No. 1722 by TRV *Seisui-maru*, we collected three specimens of *Benthoscolex*. We describe the specimens here as a new species and provide a COI sequence as a DNA barcode of the species. This is the first report of *Benthoscolex* from Japan.

## Materials and methods

Fresh specimens were collected by beam trawl from the Sea of Kumano, Japan (34°00.992'N to 33°55.258'N, 136°27.720'E to 136°26.650'E) from 487–596 m depth. The live specimens were fixed in 70% ethanol. After preservation, these specimens were observed with a Nikon SMZ1500 dissecting microscope and OLYMPUS BX51 compound microscope, and photographed with a Nikon D5200 digital camera. All of the material has been deposited in the National Museum of Nature and Science, Tsukuba (**NSMT**). We followed the morphological terminology of Barroso et al. (2017) in the taxonomic description below.

DNA extraction and sequencing for a partial region of mitochondrial cytochrome *c* oxidase subunit I (COI) gene were carried out following the method of Jimi and Fujiwara (2016). The newly obtained sequence data has been deposited in the DNA Data Bank of Japan (**DDBJ**).

## Systematics

### Family Amphinomidae Lamarck, 1818

[Japanese name: umikemushi-ka]

#### Genus Benthoscolex

Taxon classificationAnimaliaAmphinomidaAmphinomidae

Horst, 1912

##### Diagnosis.

Body fusiform, flat. Eyes absent. Caruncle consisting of three ridges without ornamentation. Branchiae absent at least in first five chaetigers, dendritically branched. Dorsal and ventral cirri occur singly on the notopodium and neuropodium.

#### Benthoscolex seisuiae sp. n.

Taxon classificationAnimaliaAmphinomidaAmphinomidae

http://zoobank.org/BC2C42AA-5761-44F6-89AE-F1D2B7260BBC

**Figure 1.**
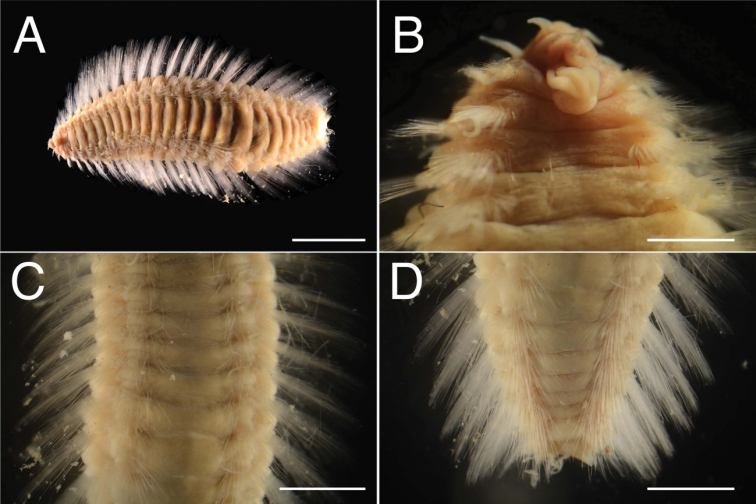
*Benthoscolex seisuiae* sp. n., holotype (NSMT-Pol H-676). **A** whole body, dorsal view **B** anterior end, dorsal view **C** median body, dorsal view **D** posterior end, dorsal view. Scale bars: 5 mm (**A**); 1 mm (**B**); 3 mm (**C–D**).

**Figure 2.**
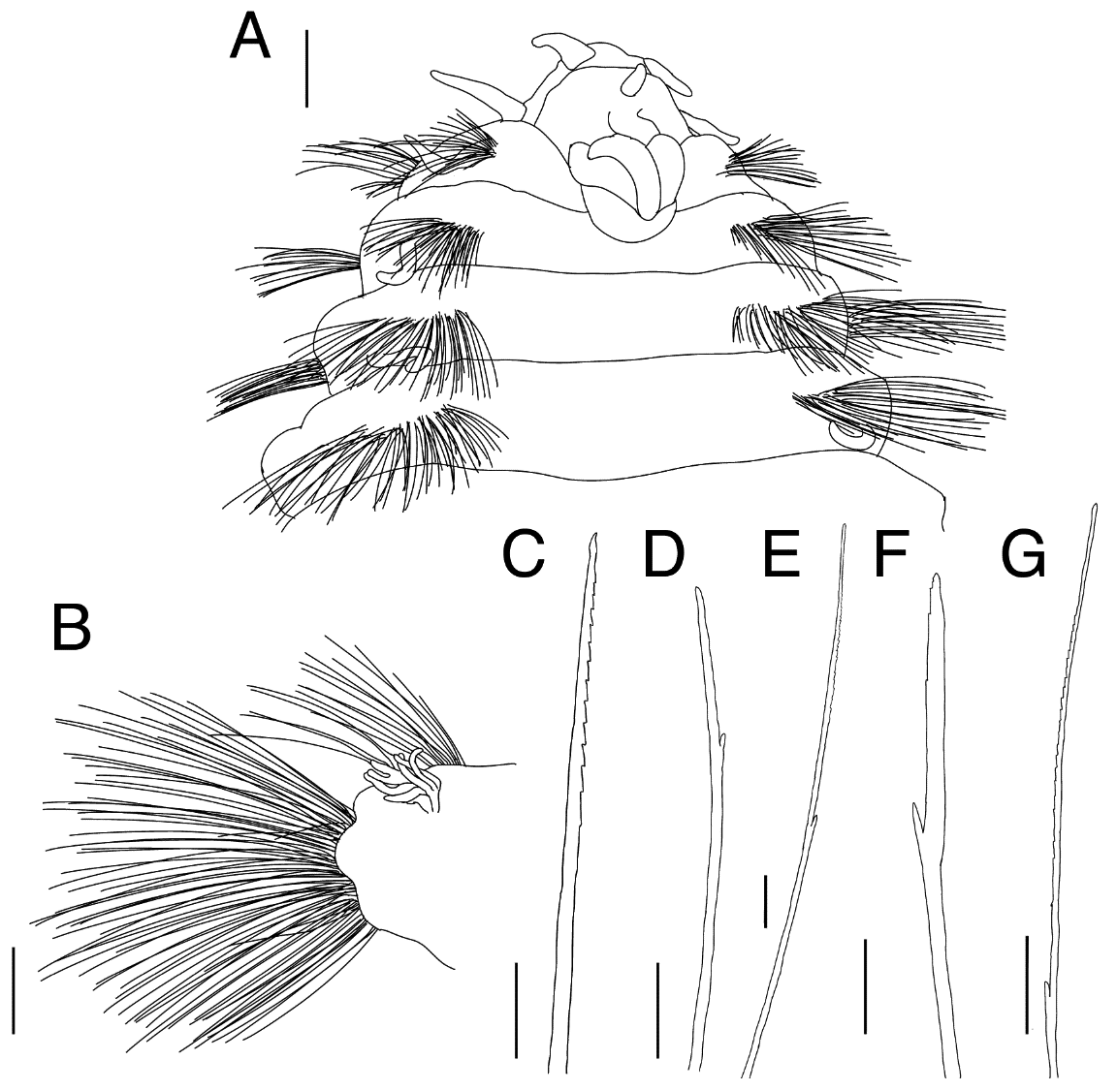
*Benthoscolex seisuiae* sp. n., holotype (NSMT-Pol H-676). **A** anterior end, dorsal view **B** left parapodium of chaetiger 15, posterior view **C** harpoon notochaeta **D** bifurcate notochaeta with short tip **E** bifurcate notochaeta with long tip **F** bifurcate neurochaeta with short tip **G** bifurcate neurochaeta with long tip. Scale bars: 1 mm (**A–B**); 100 μm (**C–G**).

Figs 1 , 2 [New Japanese name: Seisui-mitsu-one-umikemushi]

##### Material examined.

Holotype: NSMT-Pol H-676, 21 mm long, 5 mm wide (without chaetae, at widest chaetiger), 29 chaetigers, female, the Sea of Kumano, 487–596 m depth, 8 November 2017, collected by NJ (left parapodium of chaetiger 15 was dissected for DNA extraction). Paratypes: NSMT-Pol P-677, two specimens, 19–28 mm long, 4–7 mm wide (without chaetae, at widest chaetiger), 29 chaetigers, female, collection data is the same as that of the holotype.

##### Sequence.

LC360809, COI gene, 507 bp, determined from holotype.

##### Description.

Body flat, tapered in anterior and posterior regions, whitish both in life and after fixation; pair of brown longitudinal lines on ventral middle line; no pigmentation on dorsal surface (Fig. 1A). Body surface smooth.

Prostomium triangular; eyes absent. Pairs of lateral antennae and palps present, conical, smooth; palps 1.8 times as long as lateral antennae. Median antenna present, conical, as long as lateral antennae (Figs 1B, 2A). Caruncle consists of three longitudinal ridges, without ornamentation, extends to chaetigers 1–2 (depending on fixation), unattached in posterior part (Fig. 2A). Mouth composed of chaetigers 1–2. Pharynx eversible with black pigmentation.

Parapodia biramous, notopodia and neuropodia clearly separated (Fig. 2B). Dorsal and ventral cirri occur singly on notopodium and neuropodium, conical, whitish, arising from body wall, present in all chaetigers. Branchiae present on chaetiger 6 or 7 and succeeding posterior chaetigers: anterior ones simple, conical lobes; gradually increasing in number and size posteriorly (Fig. 1B, C), branched from base; filaments digitiform, 8–10 filaments per branchia in middle body chaetigers, 15–18 filaments per branchia in posterior chaetigers; branchiae in posterior chaetigers differ in size between specimens, but never reaching to tip of notochaetae (Fig. 1D).

Notochaetae contain three types: i) harpoon chaetae, with serrations limited to one side (Fig. 2C); ii) bifurcate chaetae with weakly serrated or non-serrated short tip (Fig. 2D); iii) bifurcate chaetae, with long serrated tip (Fig. 2E). Neurochaetae contain two types: i) bifurcate chaetae, with weakly serrated or non-serrated short tip (Fig. 2F); ii) bifurcate chaetae, with long serrated tip (Fig. 2G). Neurochaetae longer than notochaetae.

Anus opening dorsally on terminal chaetiger; anal papilla absent (Fig. 1D).

##### Etymology.

The species is named after the TRV *Seisui-maru*. The type specimens from the Sea of Kumano were collected by beam trawl gear of the ship. The specific name is a noun in the genitive case.

##### Confirmed distribution.

Only known from the type locality, the Sea of Kumano, Japan, 487–596 m depth.

##### Remarks.

*Benthoscolex seisuiae* sp. n. can be discriminated from *B. coecus* and *B. cubanus* by the following features: i) palps 1.8 times as long as lateral antennae (vs. same length as lateral antennae in *B. cubanus*; 2.0 times as long as lateral antennae in *B. coecus*), and ii) branchiae do not reach to tip of notochaetae (vs. extending beyond tip of notochaetae in *B. coecus*; they also do not reach to tip of notochaetae in *B. cubanus*). In addition, the tip of the bifurcate neurochaetae is reportedly serrated in *B. cubanus*, whereas it is only weakly serrated, or not serrated at all, in *B. seisuiae* sp. n., although chaetal serration is known to be variable in *Eurythoe* (Barroso and Paiva 2007).

**Figure 3.**
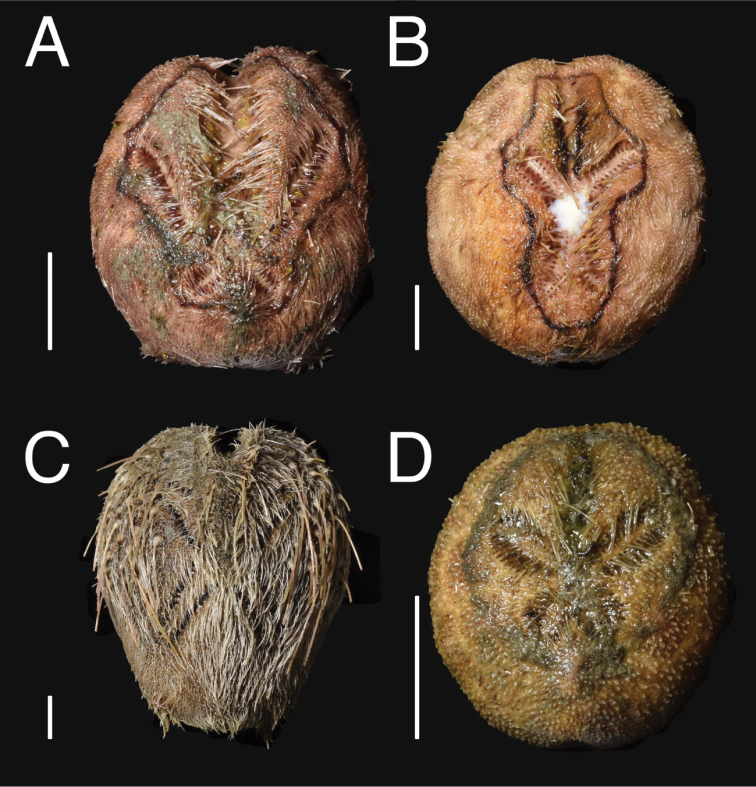
Four of the five irregular sea urchin species that were contained in the same haul with *Benthoscolex seisuiae* sp. n., aboral view. **A** *Brisaster latifrons* (A. Agassiz, 1898), NSMT E-10723 **B** *Brissopsis luzonica* (Gray, 1851), NSMT E-10721 **C** *Lovenia gregalis* Alcock, 1893, NSMT E-10719 **D** *Schizaster* sp., NSMT E-10725. Scale bars 1 cm.

*Benthoscolex cubanus* is reported to be endocommensal in the body cavity of the bathyal irregular sea urchin *Heterobrissus hystrix* (A. Agassiz, 1880) (Hartman 1942; Emson et al. 1993). *Benthoscolex seisuiae* sp. n. was collected by a beam trawl and found free living. In the same haul, 49 specimens representing five species of irregular sea urchins [*Brisaster latifrons* (A. Agassiz, 1898) (n = 13, NSMT E-10723–10724), *Brissopsis luzonica* (Gray, 1851) (n = 6, NSMT E-10721–10722), *Brissopsis* sp. (n = 1, NSMT E-10727), *Lovenia gregalis* Alcock, 1893 (n = 22, NSMT E-10719–10720), *Schizaster* sp. (n = 7, NSMT E-10725–10726)] were present and some were broken in the net. However, examination of body cavity in all but one specimen (used for species identification and photography, Fig. 3) for each species revealed no commensal *Benthoscolex* worms (A. Ogawa pers. obs.); *Brissopsis* sp. was not examined because it was represented by only one specimen. Therefore, whether the new species is also endocommensal in sea urchins or not cannot be ascertained at the moment. Future studies are required to confirm the present observations of a free-living lifestyle in the new species.

### Key to species of *Benthoscolex* Horst, 1912

**Table:** 

1	Posterior branchiae extend beyond tip of notochaetae	***B. coecus* Horst, 1912**
–	Posterior branchiae do not reach to tip of notochaetae	**2**
2	Palps 1.8 times as long as lateral antennae	***B. seisuiae* sp. n.**
–	Palps as long as lateral antennae	***B. cubanus* Hartman, 1942**

## Supplementary Material

XML Treatment for Benthoscolex

XML Treatment for Benthoscolex seisuiae

## References

[B1] Barroso R Paiva PC (2007) Amphinomidae (Annelida: Polychaeta) from Rocas Atoll, northeastern Brazil. Arquivos do Museu Nacional, Rio de Janeiro 65(3): 357–362.

[B2] Barroso R Ranauro N Kudenov JD (2017) A new species of *Branchamphinome* (Annelida: Amphinomidae) from the South-western Atlantic, with an emendation of the genus. Journal of the Marine Biological Association of the United Kingdom 97(5): 835–842. https://doi.org/10.1017/S0025315417000054

[B3] Borda E Kudenov JD Bienhold C Rouse GW (2012) Towards a revised Amphinomidae (Annelida, Amphinomida): description and affinities of a new genus and species from the Nile Deep-sea Fan, Mediterranean Sea. Zoologica Scripta 41: 307–325. https://doi.org/10.1111/j.1463-6409.2012.00529.x

[B4] Emson RH Young CM Paterson GLJ (1993) A fire worm with a sheltered life: studies of *Benthoscolex cubanus* Hartman (Amphinomidae), an internal associate of the bathyal sea-urchin *Archeopneustes hystrix* (A. Agassiz, 1880). Journal of Natural History 27: 1013–1028. https://doi.org/10.1080/00222939300770641

[B5] Fauvel P (1953) AnnelidaPolychaeta. In: Sewell RBS (Ed.) The Fauna of India, including Pakistan, Ceylon, Burma and Malaya. The Indian Press Ltd., Allahabad, 507 pp.

[B6] Hartman O (1942) Report on the Scientific Results of the Atlantis Expeditions to the West Indies under the joint auspices of the University of Havana and Harvard University. The Polychaetous Annelida. Memorias de la Sociedad Cubana de Historia Natural 16(2): 89–104.

[B7] Horst R (1912) Polychaeta Errantia of the Siboga Expedition. Part 1, Amphinomidae. Siboga-Expeditie 1899–1900 24a: 1–43.

[B8] Imajima M (2001) Deep-sea benthic polychaetous annelids of Tosa Bay, southwestern Japan. National Science Museum Monographs 20: 31–100.

[B9] Imajima M (2005) Deep-sea benthic polychaetous annelids from around Nansei Islands. National Science Museum Monographs 29: 37–99.

[B10] Imajima M (2006) Polychaetous annelids from Sagami Bay and the Sagami Sea, Central Japan. Memoirs of the National Science Museum 40: 317–408.

[B11] Imajima M (2011) Polychaetous annelids collected from Sagami Bay toward the Ogasawara islands, Japan. Memoirs of the National Museum of the Natural Science 47: 145–218.

[B12] Jimi N Fujiwara Y (2016) New species of *Trophoniella* from Shimoda, Japan (Annelida, Flabelligeridae). ZooKeys 614: 1–13. https://doi.org/10.3897/zookeys.614.8346 10.3897/zookeys.614.8346 PMC5027653 27667929

[B13] Monro CCA (1937) Polychaeta. The John Murray Expedition 1933–34, Scientific Reports, Zoology 4(8): 243–321.

[B14] Salazar-Vallejo SI (1999) Polychaetes (Polychaeta) in the Muséum National d’Histoire Naturelle collected mainly during ORSTOM cruises. http://www.ecosur-qroo.mx/Bentos/wbentos/Documentos/Polychaetes ORSTOM.doc

[B15] Sun Y Li X (2017) A new genus and species of bristle worm from Beibu Gulf, South China Sea (Annelida, Polychaeta, Amphinomidae). ZooKeys 708: 1–10. https://doi.org/10.3897/zookeys.708.12967 10.3897/zookeys.708.12967 PMC5674149 29118631

[B16] Verdes A Simpson D Holford M (2017) Are fireworms venomous? Evidence for the convergent evolution of toxin homologs in three species of fireworms (Annelida, Amphinomidae). Genome Biology and Evolution 10(1): 249–268. https://doi.org/10.1093/gbe/evx279 10.1093/gbe/evx279 PMC5778601 29293976

[B17] Wehe T Fiege D (2002) Annotated checklist of the polychaete species of the seas surrounding the Arabian Peninsula: Red Sea, Gulf of Aden, Arabian Sea, Gulf of Oman, Arabian Gulf. Fauna of Arabia 19: 7–238.

